# Association between overtime work hours and preventive dental visits among Japanese workers

**DOI:** 10.1186/s12889-020-10107-7

**Published:** 2021-01-07

**Authors:** Yoshikazu Harada, Tomohisa Nagata, Masako Nagata, Arisa Harada, Ryoichi Oya, Koji Mori

**Affiliations:** 1 Department of Dentistry and Oral Surgery, University Hospital of Occupational and Environmental Health, Kitakyushu, Japan; 2 Department of Pathology, School of Medicine, University of Occupational and Environmental Health, Kitakyushu, Japan; 3 Department of Occupational Health Practice and Management, Institute of Industrial Ecological Sciences, University of Occupational and Environmental Health, 1-1 Iseigaoka, Yahatanishi-ku, Kitakyushu, 807-8555 Japan; 4 Department of Occupational Medicine, School of Medicine, University of Occupational and Environmental Health, Kitakyushu, Japan

**Keywords:** Overtime work, Preventive dental visits, Oral health, Workplace

## Abstract

**Background:**

This study aimed to examine the association between overtime work and the frequency of preventive dental visits among workers in Japan.

**Methods:**

A self-administered questionnaire was completed by 14,847 daytime-workers. We used a logistic regression model stratified by sex and age and adjusted for marital status, occupation, education, and oral status to investigate the association between overtime work hours and the frequency of preventive dental visits.

**Results:**

In total 1037 men (9.3%) and 511 women (13.9%) attended quarterly preventive dental visits, and 2672 men (23.9%) and 1165 women (31.8%) attended annual preventive dental visits. Overtime work was statistically significantly associated with quarterly preventive dental visits among men aged 50–59 years, with adjusted odds ratios (aOR) and 95% confidence intervals (CI) of 0.73 (0.56–0.95), 0.75 (0.54–1.04), and 0.55 (0.34–0.90) for < 20, 20–39, and ≥40 h overtime/month, respectively. No such trends were observed for men aged < 50 years and women of all ages. Overtime work of < 20, 20–40, and ≥40 h overtime/month was statistically significantly associated with annual preventive dental visits among men aged 40–49 years (aOR [95%CI]: 0.76 [0.61–0.95], 0.84 [0.65–1.09], and 0.72 [0.51–1.00], respectively) and 50–59 years (aOR [95%CI]: 0.75 [0.61–0.91], 0.76 [0.59–0.97], and 0.63 [0.45–0.88], respectively). No such trends were observed in men < 40 years and women of all ages.

**Conclusions:**

Our study revealed associations between overtime and preventive dental visits among male workers aged in their 40s and 50s.

**Supplementary Information:**

The online version contains supplementary material available at 10.1186/s12889-020-10107-7.

## Background

Periodontitis and caries are the most common causes of permanent tooth loss [1–4]. Permanent tooth loss is associated with a reduction in intraoral function, and an increased risk for depression and cognitive decline [5, 6]. In addition, periodontitis has been identified as a risk factor for systemic diseases such as diabetes and coronary heart disease [7–9].

Previous studies reported that overtime work increased the risk for developing diseases such as coronary heart disease, stroke, diabetes, and mental health disorders [9–13]. Recently, overtime work has been identified as a risk factor for increased morbidity of dental diseases, such as caries and periodontitis [4, 9]. A survey of medical doctor trainees showed 71.4% had delayed or missed preventive dental examinations, with the main reason being lack of time because of work commitments [14].

Preventive dental visits to maintain good oral condition (e.g., check-ups, professional teeth cleaning, and oral hygiene advice) has beneficial effects for oral health, and poor oral hygiene is a major risk factor for periodontitis and caries. Professional teeth cleaning combined with oral hygiene advice results in a reduction in plaque and gingival bleeding [15]. In addition, dental visits reduce the incidence of periodontitis [16]. Women and older individuals are more likely to attend preventive dental visits [17]. Highly educated people also have more frequent dental visits for check-ups than poorly educated people [18]. Research suggests that overtime workers have fewer opportunities to visit hospital for general medical treatment than those that do not work overtime [19]. However, if opportunities for preventive dental visits decrease, the morbidity associated with these diseases may increase. In addition, an increase in periodontitis may influence the increase of other diseases such as coronary heart disease.

A previous cross-sectional study examined the association between working hours and use of preventive health services [20]. Individuals working long hours (> 60 per week) were significantly less likely to attend dental check-ups and cancer screening (e.g., mammograms) than those working shorter hours. Factors contributing to these results were time barriers to making appointments for screening visits and time barriers to keeping any appointments made. Another study revealed that 71% of participants reported delaying or skipping preventive dental examinations because of lack of time to schedule and attend appointments [14]. However, it is difficult to apply those results more generally because the sample size in that study was small and all participants were medical doctors.

The impact of visits to a dental clinic for preventive purposes (rather than for dental examinations) has not been examined. Therefore, we conducted the present study to examine the association between overtime work and preventive dental visits among men and women in different age groups. Preventive dental visits refer to visits to a dental clinic for preventive purposes, rather than for a set dental check-up.

## Methods

This cross-sectional study included participants from six companies in Japan (five manufacturing companies and one information and communications company). Participating companies did not conduct dental examinations. A self-administered questionnaire was disseminated to 32,026 workers across the six companies between July and October 2017. Participants received an explanation about the study and were informed that completion of the self-administered questionnaire was voluntary. The number of participants (response rate) from each of the six companies was 5728 (81.4%), 4083 (71.0%), 3943 (43.3%), 2302 (72.7%), 709 (11.7%), and 418 (46.5%). This gave a total of 17,183 participants (overall response rate: 53.7%).

The questionnaire collected information on personal characteristics, such as educational background, working conditions (working pattern [daytime, shift work, night shift and semi-night shift], mean overtime hours per month), preventive dental visits, and oral status (see Additional file 1). For the purpose of this study, 1674 shift workers (1580 shift work, 33 night shift, 28 semi-night shift, and 33 with missing data) were excluded to eliminate the effect of shift work on the frequency of preventive dental visits. Workers aged 60 years and over (*n*=662) were also excluded from the study population because working conditions in Japan change significantly for those over age 60 years. Therefore, 14,847 workers who provided valid responses were included in the present analysis.

This study used a web-based survey. The study design was explained to all employees and employers via email, the company’s intranet homepage, or the company’s occupational health and safety committee. Employees could freely choose whether to participate in this study. Employees’ responses to the questionnaire were not disclosed to their employers. The research protocol was approved by the Ethics Committee of Medical Research, University of Occupational and Environmental Health, Japan (H26–026).

### Explanatory variable: mean overtime work hours per month

The item assessing participants’ overtime was: “What was your mean number of overtime work hours per month in the last 6 months? Please choose the most applicable option (include working hours on holidays; do not include commuting time).” There were 12 response options: 0, < 10, 10–19, 20–29, 30–39, 40–49, 50–59, 60–69, 70–79, 80–89, 90–99, and ≥100 h. Mean overtime hours were grouped into four categories: 0, < 20, 20–39, and ≥40 h.

### Outcome: frequency of preventive dental visits

A Likert scale question with five levels was used to assess the frequency of preventive dental visits. Participants were asked “Are you currently visiting a dental clinic for prevention?” Response options were: “more than once every 3 months,” “once every 6 months,” “once a year,” “sometimes,” and “never.” A previous study reported that dental visits at an interval of 3 months [15, 21] or more than once per year [16] prevent periodontitis. We set two outcomes (binary variables). The first outcome was “more than once every 3 months” (yes or no), and this was classified as “quarterly preventive dental visits.” The second outcome was annual visits for prevention (yes or no), which included “more than once every 3 months,” “once every 6 months,” and “once a year.” This outcome was categorized as “annual preventive dental visits.”

### Covariates

Age was considered a continuous variable and categorized as ≤29, 30–39, 40–49, and 50–59 years. The categories for occupation (clerk, sales, research and development, engineer, production line and engineer, others) were taken from the human resource data of the participating companies. Marital status was classified into four groups: married, unmarried (single), unmarried (living with family and relatives), and divorced/bereaved. Responses were categorized as married, single/unmarried, and divorced/bereaved. Educational background and oral health status are thought to affect the dental visit behavior [18]. Educational background was categorized as “junior high school or high school,” “junior college, technical school, or high professional school,” “college,” or “postgraduate.” To understand oral health status, we asked participants: “During the past month, have you had any dental problems (such as toothache)?” (yes or no). We defined this variable as “dental problems.”

### Statistical analysis

We calculated the proportions of participants who made quarterly preventive dental visits and annual preventive dental visits for each subgroup (age, occupation, marital status, education, dental problems, and overtime work hours per month) stratified by sex. Chi-square tests were performed to evaluate associations in each category.

Logistic regression analysis was used to examine the association between overtime work hours and preventive dental visits, stratified by sex and age because the frequency of regular dental visits is high for women and older age groups in Japan [17]. “Quarterly preventive dental visits” and “annual preventive dental visits” were set as the outcome variables. “Overtime work hours” was set as an exposure variable. Adjusted odds ratios (aOR) and 95% confidence intervals (CI) were calculated, with adjustment for factors that may be associated with preventive dental visits (occupation, marital status, educational background, and dental problems) [18]. Workers with dental problems may be more likely to attend preventive dental visits than workers without dental problems because they may take preventive measures when visiting a dentist for treatment. Therefore, we performed an additional sensitivity analysis involving workers without dental problems. Statistical significance was set at *p*< 0.05. All analyses were conducted using IBM SPSS 25.0 software (SPSS Inc., Chicago, Illinois) and Stata 16.0 software (StataCorp. Texas, USA).

## Results

Overall, 11,179 men (75.3%) and 3668 women (24.7%) responded to the questionnaire (Table 1). The age distribution (≤29, 30–39, 40–49, and 50–59 years) was 8, 21, 37, and 34% in men and 20, 25, 35, and 20% in women, respectively. The majority of men were married (79%), whereas the number of single and married respondents was almost the same for women. Overall 6% of respondents (both men and women) had dental problems.

**Table 1:** Demographic characteristics of 14,847 respondents from six companies

	Men		Women	
	N	%	N	%
Total	11,179	75.3	3668	24.7
Age, years				
≦29	893	8.0	716	19.5
30–39	2338	20.9	932	25.4
40–49	4177	37.4	1298	35.4
50–59	3771	33.7	722	19.7
Occupation				
clerk	3388	30.3	1385	37.8
sales	4291	38.4	861	23.5
research and development	1428	12.8	820	22.4
engineer	276	2.5	86	2.3
production line and engineer	1567	14.0	380	10.4
others	228	2.0	135	3.7
missing	1	0.0	1	0.0
Marriage status				
Married	8794	78.7	1779	48.5
Single	2085	18.7	1680	45.8
Divorce or bereavement	261	2.3	179	4.9
Missing	39	0.3	30	0.8
Education (graduate status)				
Junior high school or high school	1638	14.7	913	24.9
Junior college or technical school or high professional school	464	4.2	695	18.9
College	6093	54.5	1354	36.9
More than graduate school	2945	26.3	680	18.5
Missing	39	0.3	26	0.7
Dental problems				
No	10,264	91.8	3410	93.0
Yes	615	5.5	221	6.0
Missing	300	2.7	37	1.0
Overtime work hours				
None	1301	11.6	643	17.5
< 20	6266	56.1	2302	62.8
20≦, < 40	2765	24.7	596	16.2
40≦	813	7.3	105	2.9
Missing	34	0.3	22	0.6

Overtime work hours: average number of overtime work hours in a month

Table 2 shows the association between preventive dental visits and demographic characteristics excluding those with missing data (*n*=2) for outcomes. In total, 1037 men (9.3%) and 511 women (13.9%) attended quarterly preventive dental visits, and 2672 men (23.9%) and 1165 women (31.8%) attended annual preventive dental visits. In any analysis of annual and quarterly preventive dental visits, the proportion of respondents attending preventive dental visits tended to increase as age increased for both men and women. In addition, the proportion of respondents attending preventive dental visits decreased as working hours increased. Men and women with dental problems tended to proactively visit a dental clinic more often.

**Table 2:** Distribution of preventive dental visits according to sex among 14,845 workers, excluding missing data for preventive dental visits (*n* = 2)

		Quarterly preventive dental visit						Annual preventive dental visit					
		Men			Women			Men			Women		
		N	%	*p* value	N	%	*p* value	N	%	*p* value	N	%	*p* value
Age, years	≤29	40	4.5	< 0.001	67	9.4	< 0.001	149	16.7	< 0.001	157	21.9	< 0.001
	30–39	178	7.6		142	15.2		545	23.3		345	37.0	
	40–49	382	9.1		154	11.9		969	23.2		410	31.6	
	50–59	437	11.6		148	20.5		1009	26.8		253	35.0	
Occupation													
	clerk	322	9.5	0.056	193	13.9	0.898	821	24.2	< 0.001	447	32.3	0.031
	sales	427	10.0		128	14.9		1116	26.0		273	31.7	
	research and development	126	8.8		109	13.3		356	24.9		280	34.1	
	engineer	23	8.3		9	10.5		49	17.8		15	17.4	
	production line and engineer	123	7.9		55	14.5		285	18.2		116	30.5	
	others	16	7.0		17	12.6		45	19.7		34	25.2	
Marriage status	Married	845	9.6	0.069	243	13.7	0.403	2212	25.2	< 0.001	593	33.3	0.027
	Single	167	8.0		234	13.9		403	19.3		498	29.6	
	Divorce or bereavement	22	8.4		31	17.3		49	18.8		65	36.3	
	Missing	3	7.9		3	10.0		8	21.1		9	30.0	
Education (graduate status)	Junior high school or high school	138	8.4	0.024	127	13.9	0.896	300	18.3	< 0.001	262	28.7	0.020
	Junior college or technical school or high professional school	38	8.2		93	13.4		83	17.9		206	29.6	
	College	613	10.1		196	14.5		1568	25.7		461	34.0	
	More than graduate school	246	8.4		92	13.5		717	24.3		229	33.7	
	Missing	2	5.3		3	11.5		4	10.5		7	26.9	
Dental problems	No	897	8.7	< 0.001	457	13.4	0.001	2410	23.5	< 0.001	1075	31.5	0.113
	Yes	118	19.2		48	21.7		219	35.6		81	36.7	
	Missing	22	7.4		6	16.2		43	14.4		9	24.3	
Overtime work hours	None	153	11.8	0.009	100	15.6	0.560	384	29.5	< 0.001	232	36.1	0.053
	< 20	576	9.2		315	13.7		1479	23.6		724	31.5	
	20≤, < 40	240	8.7		83	13.9		634	22.9		174	29.2	
	40≤	68	8.4		12	11.4		169	20.8		31	29.5	
	Missing	0	0		1	4.5		6	17.6		4	18.2	

Overtime work hours: mean overtime work hours per month in the last 6 months

*P*-values calculated by chi-square tests

Table 3 shows the results of the association between preventive dental visits and overtime work hours, stratified by sex and age. Overtime work had a statistically significant association with quarterly preventive dental visits among men aged 50–59 years, with aORs (95%CI) of 0.73 (0.56–0.95, *p*=0.019), 0.75 (0.54–1.04, *p*=0.085), and 0.55 (0.34–0.90, *p*=0.017) for < 20, 20–40, and ≥40 h overtime/month, respectively (reference: men of the same age with no overtime). No such trends were observed in men aged < 50 years and women of all ages. Overtime work had a statistically significant association with annual preventive dental visits among men aged 40–49 years, with aORs (95%CI) of 0.76 (0.61–0.95, p=0.017), 0.84 (0.65–1.09, *p*=0.194), and 0.72 (0.51–1.00, *p*< 0.05) for < 20, 20–40, and ≥40 h overtime/month, respectively (reference: men of the same age with no overtime). Similar results were observed for men aged 50–59 years, with aORs (95%CI) of 0.75 (0.61–0.91, *p*=0.004), 0.76 (0.59–0.97, *p*=0.028), and 0.63 (0.45–0.88, *p*=0.007) for < 20, 20–40, and ≥40 h overtime/month, respectively (reference: men of the same age with no overtime). No such trends were observed in men aged < 40 years and women of all ages.

**Table 3:** Logistic regression analysis of preventive dental visits according to age

		The number of participant		Quarterly preventive dental visit						Annual preventive dental visit					
				Men			Women			Men			Women		
Age	Overtime work hours	Men	Women	aOR	95%CI	*p*-value	aOR	95%CI	*p-*value	aOR	95%CI	*p-*value	aOR	95%CI	*p-*value
≦29	None	53	70	Reference			Reference			Reference			Reference		
	< 20	557	487	0.62	0.17–2.22	0.464	1.63	0.55–4.85	0.382	1.56	0.58–4.18	0.376	1.24	0.63–2.42	0.531
	20≦, < 40	214	117	0.50	0.12–2.04	0.331	2.06	0.59–7.16	0.257	1.33	0.48–3.70	0.589	1.00	0.44–2.25	0.991
	40≦	41	22	^a^ −	–	–	^a^ −	–	–	0.82	0.20–3.37	0.783	^a^ −	–	–
30–39	None	106	188	Reference			Reference			Reference			Reference		
	< 20	1450	566	0.97	0.47–1.97	0.924	0.68	0.43–1.08	0.101	0.74	0.48–1.16	0.193	0.74	0.52–1.05	0.092
	20≦, < 40	629	148	0.71	0.33–1.55	0.392	0.96	0.52–1.77	0.905	0.73	0.45–1.17	0.193	0.63	0.39–1.01	0.057
	40≦	151	30	0.64	0.23–1.72	0.373	0.60	0.19–1.91	0.387	0.63	0.35–1.15	0.131	0.62	0.26–1.44	0.263
40–49	None	509	259	Reference			Reference			Reference			Reference		
	< 20	2239	799	0.91	0.65–1.27	0.567	0.74	0.49–1.14	0.171	0.76	0.61–0.95	0.017	0.77	0.57–1.05	0.098
	20≦, < 40	1078	202	1.02	0.69–1.49	0.933	0.58	0.32–1.07	0.083	0.84	0.65–1.09	0.194	0.76	0.50–1.14	0.182
	40≦	347	38	1.04	0.64–1.67	0.882	0.71	0.23–2.18	0.551	0.72	0.51–1.00	< 0.05	1.10	0.53–2.30	0.792
50–59	None	633	126	Reference			Reference			Reference			Reference		
	< 20	2020	450	0.73	0.56–0.95	0.019	1.18	0.68–2.03	0.553	0.75	0.61–0.91	0.004	1.02	0.65–1.60	0.928
	20≦, < 40	844	129	0.75	0.54–1.04	0.085	1.06	0.54–2.06	0.869	0.76	0.59–0.97	0.028	0.89	0.51–1.54	0.668
	40≦	274	15	0.55	0.34–0.90	0.017	1.54	0.43–5.47	0.507	0.63	0.45–0.88	0.007	1.52	0.50–4.63	0.459

Overtime work hours: mean overtime work hours per month in the last 6 months

*aOR* Adjusted odds ratio (adjusted for occupation, marital status, education, and dental problems)

*CI* Confidence interval

^a^: Unable to analyze because of small numbers in each group

Table 4 shows the results of the sensitivity analysis among workers without dental problems. The results were the same in both the sex and age categories.

**Table 4:** Logistic regression analysis of preventive dental visits for workers without dental problems according to age

		The number of participants		Quarterly preventive dental visit						Annual preventive dental visit					
				Men			Women			Men			Women		
Age	Overtime work hours	Men	Women	aOR	95%CI	*p-*value	aOR	95%CI	*p-*value	aOR	95%CI	*p-*value	aOR	95%CI	*p-*value
≦29	None	41	62	Reference			Reference			Reference			Reference		
	< 20	538	465	0.54	0.15–1.90	0.336	1.20	0.40–3.60	0.745	1.91	0.65–5.61	0.241	1.14	0.57–2.28	0.720
	20≦, < 40	195	111	0.44	0.10–1.85	0.262	1.69	0.49–5.86	0.410	1.63	0.53–4.97	0.395	0.94	0.40–2.17	0.878
	40≦	34	22	^a^ −	–	–	^a^ −	–	–	1.09	0.25–4.81	0.908	^a^ −	–	–
30–39	None	100	180	Reference			Reference			Reference			Reference		
	< 20	1380	522	1.03	0.48–2.17	0.949	0.68	0.42–1.10	0.117	0.77	0.49–1.22	0.264	0.73	0.51–1.05	0.095
	20≦, < 40	583	144	0.74	0.32–1.69	0.475	1.07	0.58–1.99	0.829	0.75	0.46–1.23	0.252	0.64	0.39–1.05	0.078
	40≦	137	26	0.67	0.23–1.94	0.465	0.58	0.16–2.12	0.411	0.65	0.35–1.20	0.170	0.68	0.28–1.66	0.395
40–49	None	481	237	Reference			Reference			Reference			Reference		
	< 20	2069	741	0.98	0.68–1.40	0.910	0.77	0.49–1.19	0.238	0.77	0.62–0.97	0.028	0.76	0.55–1.04	0.082
	20≦, < 40	985	189	1.20	0.80–1.79	0.390	0.59	0.31–1.12	0.107	0.89	0.68–1.16	0.386	0.74	0.48–1.13	0.157
	40≦	325	36	1.16	0.70–1.91	0.569	0.56	0.16–1.99	0.370	0.76	0.54–1.07	0.119	1.04	0.48–2.22	0.927
50–59	None	569	105	Reference			Reference			Reference			Reference		
	< 20	1804	415	0.68	0.51–0.90	0.008	1.25	0.71–2.20	0.443	0.71	0.58–0.88	0.001	1.02	0.65–1.63	0.917
	20≦, < 40	748	119	0.68	0.48–0.97	0.032	1.11	0.55–2.23	0.766	0.75	0.58–0.96	0.025	0.90	0.51–1.59	0.711
	40≦	245	15	0.53	0.31–0.89	0.016	1.63	0.45–5.85	0.456	0.63	0.44–0.89	0.009	1.54	0.50–4.70	0.450

Overtime work hours: mean overtime work hours per month in the last 6 months

aOR: adjusted odds ratio (adjusted for occupation, marital status, and education)

CI: confidence interval

^a^: Unable to analyze because of small numbers in each group

## Discussion

Our study revealed associations between overtime and preventive dental visits among male workers aged in their 40s and 50s. This association was more pronounced in people working longer hours; however, even a small amount of overtime work (< 20 h a month) statistically significantly reduced preventive dental visits.

Some occupational factors, such as overtime work [9] and shift work [22], have been reported to increase the morbidity of dental diseases (e.g., periodontal disease); however, the direct cause has not yet been established. Preventive dental visits reduce the risk for caries and periodontal disease and have an important role in oral hygiene. The results of this study suggested that overtime work may increase dental disease morbidity in workers because of a decrease in preventive dental visits.

Overtime work had significant associations with non-attendance of preventive dental visits among male workers aged in their 40s and 50s. However, men in their 20s and 30s did not show any statistically significant association between long working hours and preventive dental visits. As men get older, they may assume more managerial roles and think more about their responsibilities at work, which may result in putting off preventive dental visits. Among the respondents in this study, the proportion of male managers was 0.1% for those aged ≤29 years and 3.3% for those in their 30s, compared with 32.3% for those in their 40s and 43.9% for those in their 50s. Previous research has shown that workers delay or skip preventive dental examinations because of lack of time or not wanting to burden their co-workers by delegating additional work [14]. Previous studies have also shown that workers who have more frequent dental visits are more likely to be absent from work [23]. In addition, many workers do not want to take time away from their jobs, and may therefore stop making preventive dental visits to avoid delaying or not completing work. However, few studies have investigated the reasons for failure to attend preventive dental visits, and more research is needed on this topic. This study also showed that there was no statistically significant association between long working hours and preventive dental visits among women of all ages. The proportion of female workers in managerial positions in this study was considerably lower than that of males (0% for those ≤29 years, 2.3% for those in their 30s, 11.6% for those in their 40s, and 10.4% for those in their 50s). However, the sample size of women in this study might not have been sufficient, and further studies with more women are needed.

The younger generation had a lower percentage of people who received preventive dental visits, although the proportion increased with age (Table 2). We also observed that women attended preventive dental visits more regularly than men. This was consistent with a previous study in the Japanese population, which reported that 6.5% of men and 9.0% of women attended 3-monthly preventive dental visits, and 31.4% of men and 39.9% of women attended annual preventive dental visits [17]. Similar sex differences were observed in the present study (quarterly preventive dental visits: men 9.6%, women 14%; annual preventive dental visits: men 37.8%, women 47.8%).

The influence of overtime work hours on preventive dental visits in men was greater for annual visits, compared with quarterly visits. A previous study found oral health literacy was associated with the frequency of using regular dental care [24]. For workers attending quarterly dental visits, longer working hours did not appear to affect their dental clinic visits.

The results of this study suggested that even a relatively small amount of overtime work is likely to increase the risk for dental disease because it reduces the frequency of preventive dental visits. We believe that improving management of working hours and reducing the amount of overtime work are important factors for promoting preventive dental visits, which play a vital role in oral health. In addition to reducing overtime hours, it is important to consider other options, such as conducting dental examinations in workplace settings [25] or allowing workers to visit dental clinics during working hours, particularly younger workers and male workers aged over 40 years.

The main strengths of this study were the large sample size and the detailed analysis of Japanese people belonging to large companies. Our analyses were classified by the frequency of preventive dental visits and stratified by sex and age and adjusted for many covariates. This study also had some limitations. First, we were unable to examine causal relationships because this was a cross-sectional study. It is difficult to assume a relationship whereby preventive dental visits affect overtime. Future longitudinal analyses using prospective cohort designs should be conducted. Second, our study used a self-administered questionnaire to collect data on overtime and frequency of preventive dental visits, and recall bias might be present. Consideration should be given to obtaining objective data on overtime in further studies, such as obtaining company personnel data. Regarding preventive dental visits, it is difficult to distinguish between dental visits for treatment and preventive dental visits in objective data for receipts in Japan. Therefore, it is necessary to verify the validity of preventive dental visits by interviewing some respondents and asking them about their specific behavior and the purpose of their dental visits. The third limitation is the possibility of selection bias because the data were from employees of six large companies. The effects of overtime work may differ in our study population from those in the general population because background factors were evened out.

## Conclusion

Our study revealed an association between overtime and preventive dental visits among male workers aged in their 40s and 50s. In addition to reduction of overtime work, access to oral health measures in the workplace will help improve oral health in for people who work full-time.

## Supplementary Information

**Additional file 1.** Self-administered questionnaire

## Data Availability

The datasets generated during and/or analyzed during the present study are not publicly available because of the personal privacy of participants, but are available from the corresponding author on reasonable request.

## Abbreviations

aOR: Adjusted odds ratio
CI: Confidence interval
