# Supplementary material for: Association between overtime work hours and preventive dental visits among Japanese workers
Source: BMC Public Health. 2021 Jan 7;21:87. doi: 10.1186/s12889-020-10107-7 (PMC7791987; doi:10.1186/s12889-020-10107-7)
Supplement: Supplementary file 1 — Additional file 1. Self-administered questionnaire [file 12889_2020_10107_MOESM1_ESM.docx]

**File name: Additional file 1**

Title of data: Self-administered questionnaire

**Part 1: Personal characteristics**

1. Sex: A. Men B. Women

2. Age:

3. Marriage status: A. Married B. Unmarried (single)

C. Unmarried (living with family and relatives) D. Divorce or bereavement

4. Education (graduate status):

A. Junior high school B. High school C. Junior college or technical school

D. High professional school E. College F. Postgraduate

**Part 2: Working conditions**

5. Working pattern: A. Daytime B. Shift work C. Night shift D. Semi-night shift

6. What was your mean number of overtime work hours per month in the last six months? Please choose the most applicable option (include working hours on holidays; do not include commuting time)

A. 0 (hours) B. <10 C. 10–19 D. 20–29 E. 30–39

F. 40–49 G. 50–59 H. 60–69 I. 70–79

J. 80–89 K. 90–99 L. ≥100

**Part 3. Preventive dental visits and oral status**

7. Are you currently visiting a dental clinic for prevention?

A. More than once every three months B. Once every six months

C. Once a year D. Sometimes E. Never

8. During the past month, have you had any dental problems (such as toothache)?

A. Yes B. No
